# Microsponge-Based Gel Loaded with Immunosuppressant as a Simple and Valuable Strategy for Psoriasis Therapy: Determination of Pro-Inflammatory Response through Cytokine IL-2 mRNA Expression

**DOI:** 10.3390/gels9110871

**Published:** 2023-11-01

**Authors:** Yasir Mehmood, Hira Shahid, Umar Inzamam ul Huq, Hamza Rafeeq, Hafiz Muhammad Bilal Khalid, Mohammad N. Uddin, Mohsin Kazi

**Affiliations:** 1 Department of Pharmaceutics, Faculty of Pharmaceutical Sciences, Government College University Faisalabad, Faisalabad P.O. Box 38000, Pakistan; 2 Riphah Institute of Pharmaceutical Sciences (RIPS), Riphah International University Faisalabad, Faisalabad P.O. Box 38000, Pakistan; 3 Department of Pharmacology, Faculty of Pharmaceutical Sciences, Government College University Faisalabad, Faisalabad P.O. Box 38000, Pakistan; hira.shahid16@yahoo.com; 4 Lahore College of Pharmaceutical Sciences, Lahore P.O. Box 54000, Pakistan; dr.umerinzamam@gmail.com; 5 Department of Biochemistry, Riphah International University, Faisalabad Campus, Faisalabad P.O. Box 38000, Pakistan; hamza.rafeeq@riphahfsd.edu.pk; 6 Department of Biochemistry, University of Agriculture Faisalabad, Faisalabad P.O. Box 38000, Pakistan; 7 College of Pharmacy, Mercer University, 3001 Mercer University Drive, Atlanta, GA 30341, USA; uddin_mn@mercer.edu; 8 Department of Pharmaceutics, College of Pharmacy, King Saud University, P.O. Box 2457, Riyadh 11451, Saudi Arabia

**Keywords:** tacrolimus, microparticles, drug delivery, permeability, psoriasis therapy

## Abstract

Tacrolimus (TL) is a topical calcineurin inhibitor immunosuppressive drug widely used to manage various skin disorders. Herein, we report a TL-loaded microsphere gel formulation with severe atopic dermatitis effects that are required to manage skin disorders. The current study adopted a modified emulsion solvent evaporation technique to synthesize TL-loaded microspheres, which were further converted into gels for skin use. Characterization of the synthesized formulation was performed by differential dynamic light scattering, scanning electron microscopy (SEM), Fourier transform infrared (FTIR) spectroscopy, X-ray crystallography, Brunauer–Emmett–Teller (BET) analysis, differential scanning calorimetry, and drug release. A Franz diffusion cell was used to study the diffusion of TL for up to 8 h at pH 6.8 and 5.5. Evaluation of cell viability was determined by MTT assay and showed higher IC50 values compared to the plain drug. RNA extraction, real-time polymerase chain reaction (RT–PCR), and reverse transcription were also performed to determine the expression levels of the anti-inflammatory cytokine IL-2. Particle size determination was performed by a zeta sizer, and the TL microsphere size was 1745 ± 70 nm with a good polydispersity (0.337 ± 0.12). The drug entrapment efficiency was also very good at 60% ± 10, and the drug release was 93.9% ± 3.5 within 8 h. An in vitro diffusion study of the formulation also showed improved permeability at both pH values (4.5 and 5.5). The findings of the hemolytic tests demonstrated that TL-MG at concentrations of 50, 100, and 200 mg/mL did not produce any hemolysis. A dose-dependent pattern of cytotoxicity was found during the cell viability assay, with an IC50 value of 787.55 ± 12.78 µg/mL. There was a significant decrease in the IL-2 level in the TL-MG group compared to the other groups. TL-MG microspheres were nontoxic carriers for tacrolimus delivery, with greater loading capacity, a significant release profile, and enhanced cellular uptake with improved permeability.

## 1. Introduction

The transdermal drug delivery system (TDDS) is one of the most promising routes of administration [1]. It is a noninvasive method of drug delivery that has been widely investigated for significantly improving the delivery of various therapeutic agents, such as analgesics, hormones, cardiovascular drugs, and drugs for central nervous systems [2,3]. TDDS is a noninvasive painless technique through which therapeutic agents are applied to intact and healthy skin and delivered into the systemic circulation. The therapeutic moiety being applied onto intact and healthy skin penetrates through the stratum corneum. During the application of drug-loaded transdermal drug delivery systems, the drug moieties initially penetrate deep into the epidermis and dermis. During the process of penetration, therapeutic agents do not accumulate in the dermal layer. After passing through the dermal layer, the moieties are then absorbed into the systemic circulation by the process of microcirculation [4]. TDDS has been reported as a painless and self-administration technique since the 1800s [5]. TDDS provides enhanced bioavailability, reduces dosing frequency, and lowers first-pass metabolism [6]. The sites of application for transdermal drug delivery systems are typically the upper outer arm, upper torso, and buttocks. The permeability of the loaded drug is mainly affected by the skin thickness, skin integrity, hydration level, pH of the absorption site, blood flow to the skin, and formulation components of the devices [7].

Microsponges are colloidal particles with a size range of 5–300 µm that have the ability to control the release of drugs for prolonged periods of time. These have been researched for biomedical applications such as targeted drug delivery, transdermal drug delivery, anticancer drug delivery, and bone substitutes [8]. The topical application of microsponges against skin conditions has been proficiently reported [9]. The particles do not penetrate deep into the skin, thus eliminating the systemic side effects of the drug [10]. However, prolonged retention of microsponges on the surface of the skin requires their incorporation into suitable vehicles such as emulgels, creams, or gels [11].

Microsponge gel formulations, when used for skin application, reduce systemic exposure and minimize local cutaneous reactions to active drugs [12]. Microsponges are made up of active agent-loaded macroporous beads. Microsponges are typically used for topical and, more recently, oral administration; when applied to the skin, they release their active ingredient in a time mode and in response to other stimuli (rubbing, temperature, pH, etc.) [12]. Using a microsponge delivery system (MDS) is an innovative and effective way to boost the potency of topically active drugs while also improving safety, extending product stability, allowing for more variation in formulation, lessening adverse effects, and enhancing aesthetic qualities. In addition, they are harmless and will not cause any harm to you or anybody else [13]. The small polymer microspheres at the heart of Microsponge Systems are capable of suspending or entrapping a wide range of substances; these microspheres can then be included in a manufactured product such as a gel, cream, liquid, or powder. The outside is often permeable, allowing for a steady outflow of chemicals [14].

Liquid–liquid suspension polymerization, quasi-emulsion solvent diffusion, oil-in-oil emulsion solvent diffusion, water-in-oil-in-water (w/o/w) emulsion solvent diffusion, lyophilization method, porogen addition method, vibrating orifice aerosol generator method, electrohydrodynamic atomization method, and ultrasound-assisted microsponge are some of the efficient methods for creating microsponges [8].

In the emulsion solvent evaporation technique, microsponges composed of Eudragit RS100 or methylcellulose slowly release the drug and are mostly not recommended for topical treatments [15]. Recently, microsponges designed with a combination of hydrophilic-hydrophobic interactions showed high drug loading for up to 8 h [16].

One of the most prevalent autoimmune disorders, psoriasis, is characterized by persistent skin irritation. Approximately 2 to 4% of the world’s population is impacted [17]. The skin of this horrible illness is flaky, scaly, and irritating. Additionally, swelling, pain, and skin lesions that are disfiguring are linked to it. The pathogenesis of psoriasis is extremely complex and dynamic. Skin trauma, stress, infections, and some drugs are possible causes. In addition, it can be brought on by a variety of environmental variables and even by a person’s genetic makeup [18]. Most topical therapies are used to address the mild to moderate symptoms that affect approximately 80% of psoriasis sufferers. Phototherapy and systemic therapies are used to treat those with severe illness. Due to its intrinsic benefits, such as higher patient compliance, localized therapeutic activity, and little systemic toxicity, topical therapy is still currently the preferred method of treatment for the management of psoriasis [19]. However, a significant downside of topical therapies is insufficient medication penetration through the scaly psoriatic skin. As a result, research on anti-psoriatic therapy still needs to focus on a carrier system that can carry a therapeutic quantity of medicine through sick tissues [18].

In 1984, the macrolide antibiotic tacrolimus (TL) was initially developed. It is a product of *Streptomyces tsukubaensis* and is employed as an immunosuppressant. The treatment of graft rejection and atopic dermatitis may benefit from using it therapeutically. When bound to the immunophilin FKBP-12 (FK506 binding protein), TL inhibits peptidyl-prolyl isomerase activity [20]. The formation of the new complex FKBP12-FK506 causes inhibition of calcineurin, which hinders T-lymphocyte signal transduction and IL-2 transcription [21]. TL has shown incomplete absorption through the oral route because of its relative bioavailability of <30% [22]. The current project aimed to synthesize a novel microsphere system for the delivery of TL for topical administration that holds good anti-inflammatory properties against psoriasis. The characterization of TL microspheres was performed by XRPD, DSC, FTIR spectroscopy, BET analysis, and SEM. Rats were used for the in vivo study to determine the anti-inflammatory properties of TL.

## 2. Results and Discussion

TL-M microspheres were synthesized using a modified emulsion solvent evaporation technique by incorporation into a polymer matrix of PVA and poloxamer. TL-M microspheres were spontaneously synthesized when the internal polymeric phase entered the external phase of the surfactant solution with constant stirring, which involved the evaporation of organic solvent and allowed the formation of spherical porous microspheres. Surfactant molecules (Tween 60) served as pore-forming agents in the external phase. The drug entrapment efficiency was 60% ± 10 when determined by HPLC.

### 2.1. Particle Size, Polydispersity Index (PDI) and Zeta Potential

**Figure 1:**
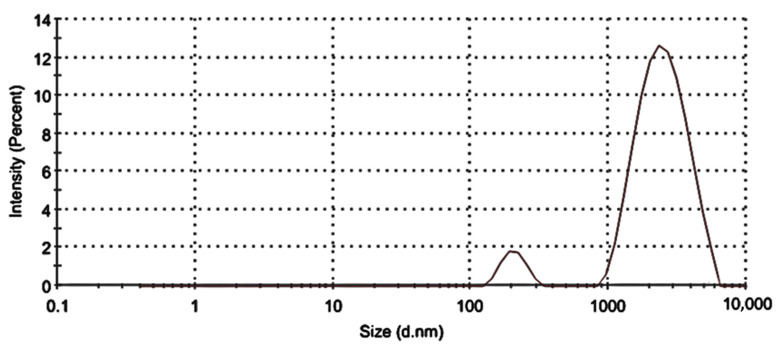
Size distribution of TL-M.

DLS was utilized to ascertain and characterize the TL-M particle sizes as well as their size distributions. Figure 1 clearly shows the presence of 2 particle fractions with sizes of ~200 nm and ~2000 nm. The DLS measured diameter was approximately 1731 nm ± 70 with a PDI of 0.337. The analysis of Microsponges’ zeta potential verified the presence of stable microsized particles, which exhibited a zeta potential of −12.13 mV. The zeta potential of a particle is a measure of both the surface charge and the stability of the particle. If the value of the zeta potential is more than 10 mV, then the particles will not cling to one another, and the system will be more stable [23,24,25].

### 2.2. FTIR Analysis

**Figure 2:**
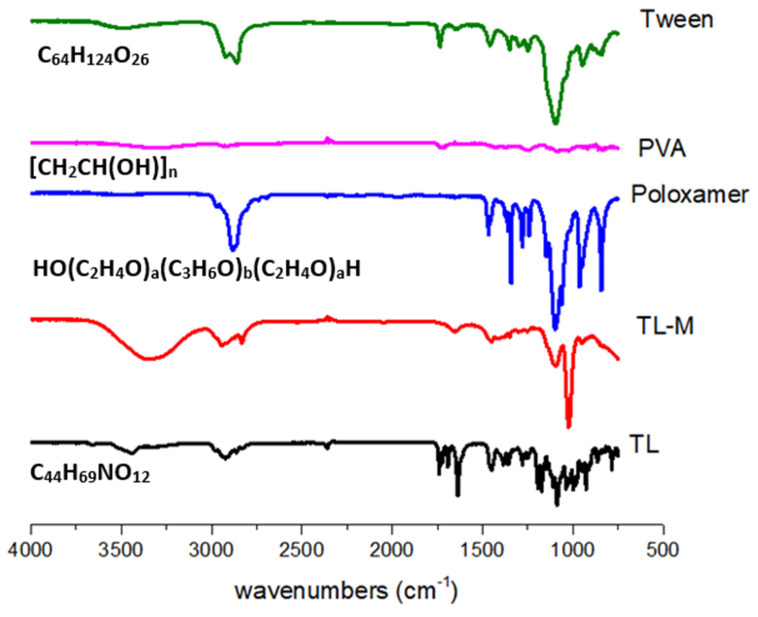
FTIR analysis of TL, TL-M, Tween 60, PVA, and poloxamer.

Figure 2 displays the FTIR spectra of TL, PVA, poloxamer, Tween 60, and TL-M. The range of 4000–600 cm^−1^ was used to scan each and every sample. The FTIR spectra of TL, shown in Figure 2, exhibited a broad band at 3390 cm^−1^. This band can be explained as being caused by the N−H stretching of primary amines. The CH_2_ frequency band can be found at 2915 cm^−1^. It is possible to witness the C=O stretching of amide-I at 1635 cm^−1^, and the N-H bend of amide-II in TL may be observed at 1540 cm^−1^. The presence of a carboxylic group was demonstrated by the appearance of a small peak at 1350 cm^−1^. In the prior literature, spectra that were comparable to this one were reported [26,27,28]. The FTIR spectrum of tween 60 (Figure 2) showed a peak at 3404 cm^−1,^ which proved the presence of COOH. Corresponding to the COOH group, another band at 2935 cm^−1^ was observed because of the methylene group. At 1609 cm^−1^, the obtained peak was related to amide-I present in the sample. At 1412 cm^−1^, the observed band was due to the deformation of OH [29]. The FTIR spectrum of the pure PVA reference sample is shown in Figure 2. It clearly reveals the major peaks associated with poly(vinyl alcohol). For instance, a broad C−H alkyl stretching band (2850–3000 cm^−1^) and typical strong hydroxyl bands for free alcohol (nonbonded -OH stretching band at 3600–3650 cm^−1^) and a hydrogen-bonded band (3200–3570 cm^−1^) can be observed [30]. The FTIR spectrum of the poloxamer depicted N−H vibrational stretching at 3300.83 cm^−1^. C=O vibrational stretching was observed at 1730.12 cm^−1^ [31,32]. FTIR spectroscopy of TL-M showed the interaction of TL with polymeric blends. The C=O stretching of amide-I can also be observed in TL-M at 1615 cm^−1^ and the N−H bending of amide-II in TL at 1520 cm^−1^, which were slightly moved but intact with low intensity. Methylene asymmetrical stretching was also observed at 2900 cm^−1^ and 1710 cm^−1^, while alkenyl stretching (C=C) of PVA moved from 1632 cm^−1^ to 1655 cm^−1^. The band shifting of the formulation components was due to the crosslinking of polymeric chains. The results showed successful interaction and entrapment of TL within polymeric microspheres, which indicated no significant drug spectrum change.

### 2.3. DSC Analysis

**Figure 3:**
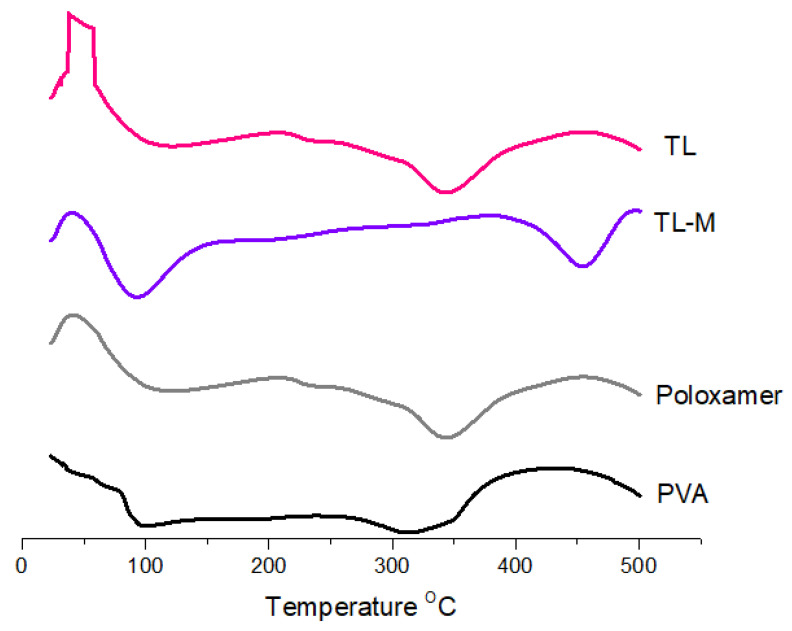
Thermal analysis of TL, TL-M, poloxamer, and PVA.

DSC was carried out using a device manufactured in the United States by Perkin Elmer. The temperature range was set to be between 25 and 500 °C, and the flow velocity was kept at 20 °C per minute [33]. The presence of moisture in the material caused the pure TL to exhibit an endothermic peak at 50 °C. In addition, two endothermal peaks were observed in the DSC curves of the TL. These peaks corresponded to the melting point of the TL, which was approximately 100 °C [33] and approximately 330 °C, respectively. On the poloxamer DSC graph, a clear somewhat exothermic peak was observed at 50 °C, and an endothermic peak was observed at 100 °C, which suggests moisture loss in the poloxamer (Figure 3). This was observed at an endothermic peak at 100 °C. In addition, the breaking of bonds in the polymeric network was shown by a distinct exothermic peak at 340.88 degrees Celsius [34]. PVA also showed endothermic peaks at 100 °C and 300 °C, which corresponded to the melting point of PVA. The endothermic peak observed at 84.9 °C in TL-M may be due to moisture loss and was shifted from 50 °C to 84.9 °C, and one more endothermic peak was observed at approximately 450.08 °C, corresponding to the melting point of TL, which was changed from 330 °C to 450.08 °C [35]. The findings from the current results suggest that tacrolimus is likely present in a molecular or amorphous state within TL-M and demonstrates greater stability compared to TL alone. However, further conclusions cannot be drawn based solely on these results.

### 2.4. Morphology Analyses

**Figure 4:**
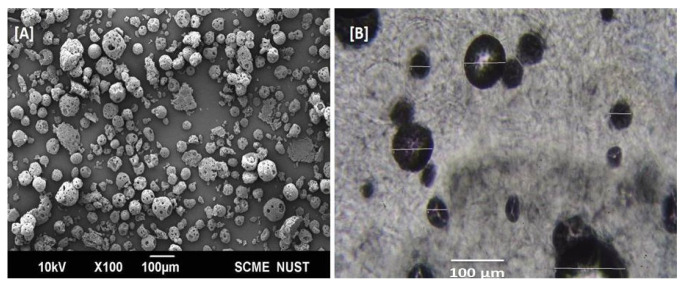
SEM images of Microsponges (**A**) and TL–MG (**B**).

Microsponges generally have high porosity, which plays an important role in the controlled release of entrapped drug particles [36]. Well-dispersed TL-M was observed by scanning electron microscopy (Figure 4A). The microsponges showed a spherical shape and porous surface (Figure 4A). The presence of the porous surface can be attributed to phase evaporation during hardening of the formulation. In Figure 4B, a compound microscopy image of TL-MG is shown.

### 2.5. Powder X-ray Diffraction Study (PXRD)

**Figure 5:**
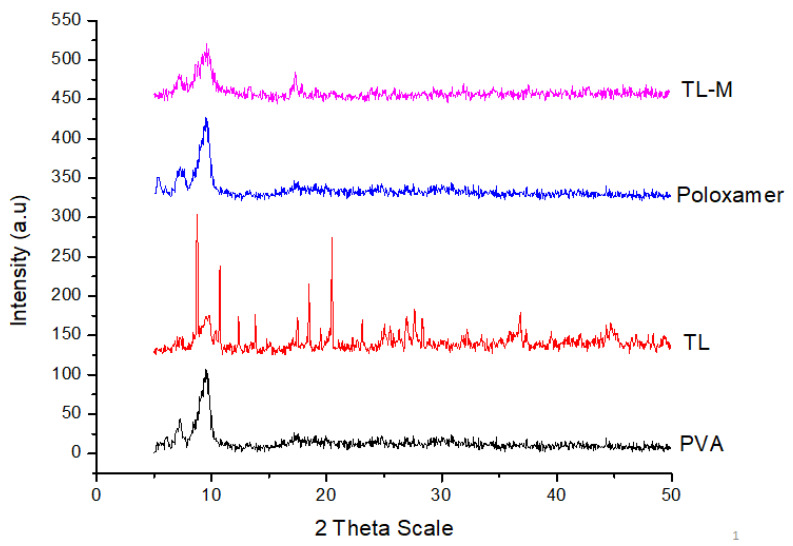
XRPD patterns of pure TL, PVA, poloxamer, and TL-M.

Figure 5 presents the PXRD patterns for pure TL, poloxamer, PVA, and TL-M. Figure 5 also includes these patterns. TL emerges in the form of sharp peaks with crystalline structures. The PXRD patterns were comparable to those of polymorphs, with large peaks observed at 22.03°, 23.82°, 26.06°, 30.35°, and 33.49° on the 2-theta scale. These peaks correspond to the crystalline tendency of TL. The poloxamer copolymer generated two distinguishable peaks at 19.2° and 24.0°. The presence of a typical semicrystalline structure was deduced from the fact that PVA displayed a maximum intensity diffraction peak at 19.8°, which corresponded to a d spacing of 4.4801 Å. On the other hand, there were no conspicuous peaks found in the Microsponges formulation, which verified the transformation of TL into an amorphous form due to the enhanced pore constriction provided by the polymer matrix [37].

### 2.6. Nitrogen Adsorption–Desorption Analysis

**Figure 6:**
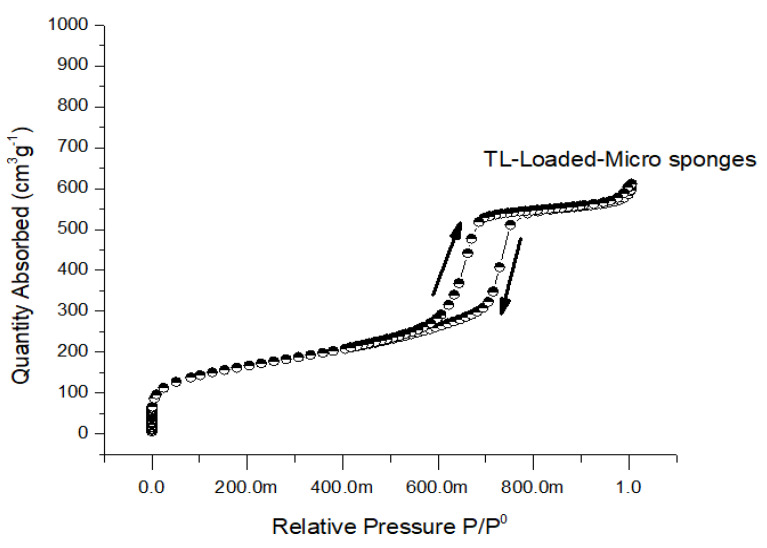
Nitrogen adsorption/desorption isotherms of TL-loaded Microsponges.

Porosity levels are often quite high in microsponges that are made using the solvent diffusion approach. BET analysis was utilized to assess the respective surface areas, pore sizes, and volumes of the unloaded and loaded surfaces [38]. The BET surface area of the unloaded microspheres was S_BET_ (m^2^/g) 6.31 ± 0.22, and the surface area of the loaded microspheres was 5.80 ± 0.09. From the results, it was noted that the minor reduction in the BET surface area of loaded Microsponges compared to unloaded Microsponges was due to adherence at the surface and entrapment in Microsponges. Similarly, the pore size of unloaded Microsponges was 3.97 ± 0.12 nm, and the pore size of loaded Microsponges was 1.94 ± 0.16, which was found to be decreased in loaded Microsponges when the obtained results were compared with unloaded Microsponges (Figure 6).

### 2.7. In Vitro Drug Release Study

**Figure 7:**
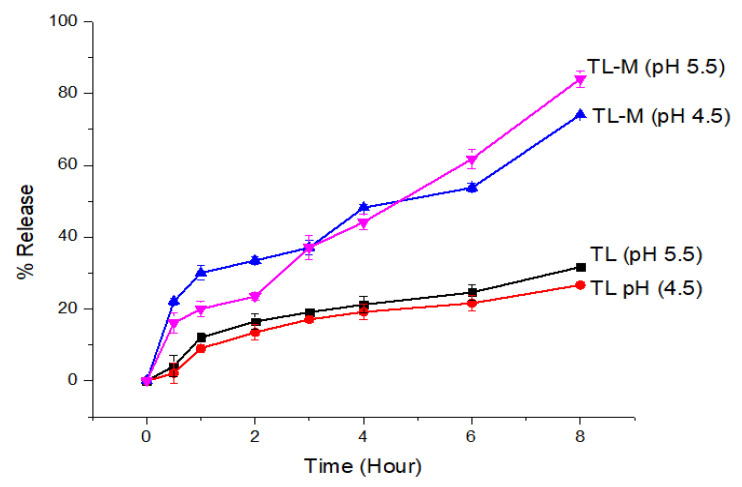
The in vitro drug release profiles of TL and TL-M were investigated using the dialysis bag membrane method (n = 3) in two different conditions: acidic buffer at pH 4.5 and phosphate buffer at pH 5.5.

To achieve an effect that lasts for an extended amount of time, the TL-M compound was designed to delay the onset of the drug’s effects for up to eight hours. Figure 7 depicts the biphasic kind of sustained-release pattern that was seen in the in vitro TL release profile obtained from TL-M. Because the pure drug release pattern was very sluggish at both pH values, TL is essentially an insoluble medication, with a water solubility of between 1 and 5 mcg/mL. It exhibited nearly the same pattern of low solubility in each of these media. The TL-M formulations initially exhibited a pattern of drug delivery that was characterized by delayed release, which was thereafter succeeded by a pattern characterized by continuous delivery of the drug. Since some of the drug moieties may only be slightly entrapped, this meant that the initial trend was virtually as high as it might have been. The sustained release pattern obtained after the initial trend was linked to slow diffusion and the release of drugs from the polymer matrix. The burst release of the formulation was approximately 23% in 2 h in acetate buffer with successive drug release of 74–84% in 8 h at pH 4.5 and 5.5.

### 2.8. Kinetic Modeling

Several dissolving models were used to investigate TL release from TL-M Microsponges. These models included the zero-order models, the first-order model, the Korsmeyer-Peppas model, the Higuchi model, and the Hixson-Crowell model. Consistent with previous studies, this study demonstrated that for up to 8 h, the optimized TL-M microspheres provided a very good match to the zero-order release model (R^2^ = 0.9429) [39]. After pores are created in the polymeric system, the rest of the mathematical model shows that the medications contained within are distributed throughout the body via diffusion [36]. Table 1 of the Korsmeyer-Peppas R^2^ data shows that the TL-M Microsponges used a super case-II transport mechanism, with water absorption and relaxation serving as the controlling variables, and it is the most suitable model for release kinetic modeling (0.9650). The microsponge API release was elucidated using the following kinetic models [39].

### 2.9. Physical Appearance, Viscosity, Spreadability and pH Determination of TL-MG

For topical applications, physical characterization of the microsphere formulation (TL-MG) was performed (Table 2). The appearance of the prepared TL-MG was clear and transparent due to Carbopol, and it was compared with a commercial product (Imunol). The TL-MG formulation had a pH of 5.5 (±0.1), which is within the acceptable range for skin care products. Previous research has indicated that gel compositions with a pH ranging from 4.5 to 5.5 are suitable for use on human skin [40,41]. At a temperature of 25 °C, the formulation had a viscosity of 8712 Cpi. The spreadability of the formulation was determined based on its hardness and compressibility when applied to glass slides. The adhesive and cohesive characteristics of the formulation and its ability to be retained on the surface depended on its hardness on the glass slide. After the addition of Carbopol gel, TL-MG demonstrated a marginal improvement in compressibility, along with a small reduction in retention time and a reconstruction of its structure, as shown in the present work.

### 2.10. In Vitro Diffusion Studies of TL-MG

**Figure 8:**
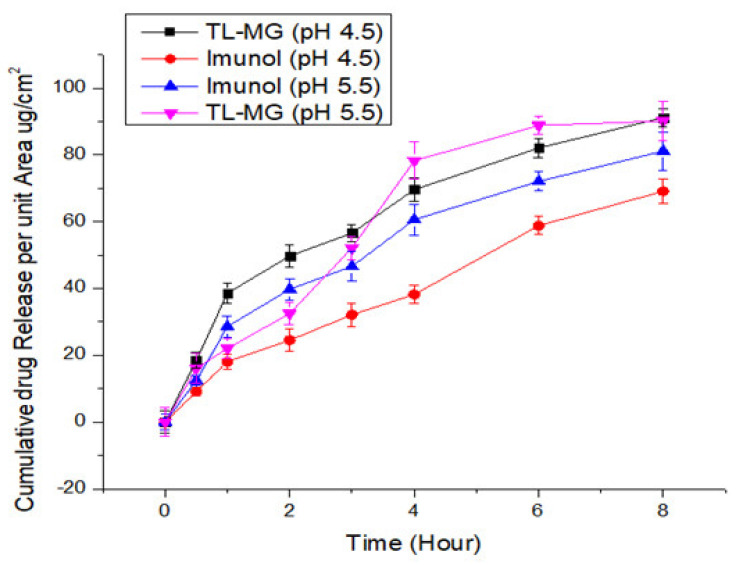
TL diffusion from TL-MG compared with Imunol.

Evaluation of the transdermal permeability of pH 4.5 and 5.5 TL-MG microspheres that were manufactured to pass through a silica membrane was the major purpose of the current investigation. The receptor cell, which was clean and dried, was filled with deaerated phosphate buffer and allowed to sit in a heated magnetic block at 37 °C for 15 min. The temperature needed to be maintained at 37 °C. The prehydrated silica membrane that was used was placed in the space that was created between the matched donor compartment and the receptor compartment. One gram of TL-MG was weighed out and placed in the internal storage compartment. A parafilm covering was placed over the openings, and then they were taped shut to avoid any direct loss of moisture. When agitating the receptor compartment, a speed of 200 revolutions per minute was utilized. At a wavelength of 220 nm, HPLC analysis was performed on a final sample that was produced using a glass syringe and had a volume of 0.5 mL. A new sample of the same volume that had been warmed was brought in so that the total amount of sample that was present in the receptor compartment could be maintained at the same level. According to the findings, there was a significant amount of TL that passed through the Strat-MTM membranes at both pH levels (4.5 and 5.5), which contributed to the improved permeability of TL-MG. The comparative results from the in vitro tests showed that the TL-MG release profile was higher regardless of the pH. The results of the in vitro permeation testing showed that the formulation had the potential to lengthen the amount of time it took for the TL to be delivered compared to the commercial product Immunol (Figure 8). Based on these data, this method has great potential for use in drug delivery systems.

### 2.11. Hemolysis Investigations

**Figure 9:**
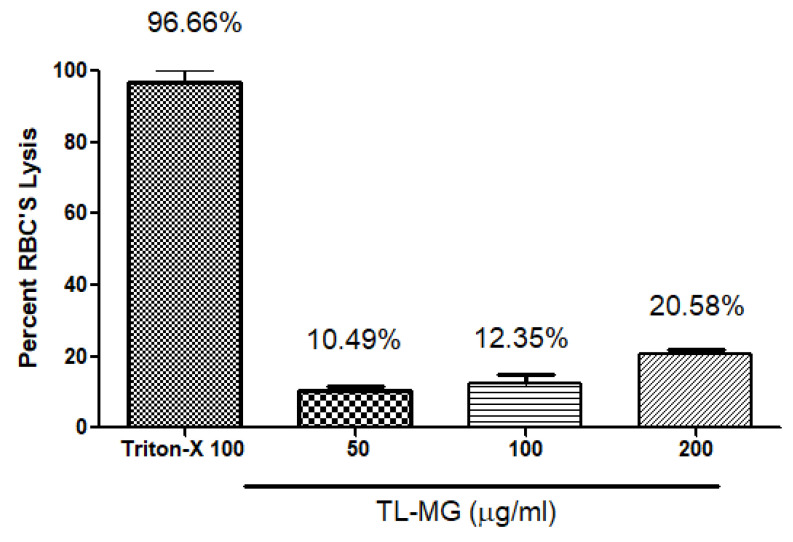
The compatibility of TL-MG with blood.

Hemolytic tests were performed to evaluate whether TL-MG was compatible with blood. The findings of the hemolytic tests (shown in Figure 9) demonstrated that TL-MG at concentrations of 50, 100, and 200 mg/mL did not result in any hemolysis being produced. Even at high concentrations, such as 200 mg/mL, there was no evidence of hemolysis, and blood cells showed a significant degree of tolerance to the complex. The findings of this investigation revealed that the concentration had an effect on the viability of the cells, and the fact that the cells survived incubation with TL-MG provided evidence that it is compatible with blood. Figure 9 shows the maximum blood hemolysis percentage against 50, 100, and 200 mg/mL (10.49 ± 2.1, 12.35 ± 3.8, and 20.58 ± 5.2%).

### 2.12. In Vitro Cell Viability Testing

**Figure 10:**
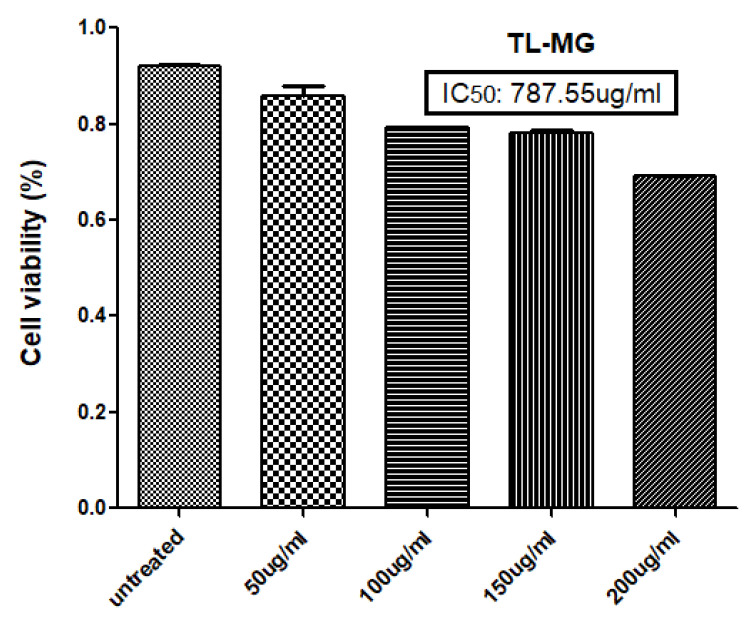
Percent toxicity induced at 50, 100, 150, and 200 µg/mL TL-MG.

The results of the MTT experiment conducted on the human hepatocellular cell line (HepG2) indicate that at concentrations below 200 µg/mL of the TL-MG formulation, the cell viability was lower. The experiment involved five different concentrations (50, 100, 150, and 200 µg/mL) of TL-MG, as depicted in Figure 10. It would appear that even at the maximum dose, this cell toxicity is not significant. The TL-MG concentration in water is virtually harmless and is compatible with biological systems. The dose-dependent pattern of cytotoxicity was demonstrated by the fact that the percentage of toxicity that was induced by TL-MG at concentrations of 50, 100, 150, and 200 µg/mL was 19.22 ± 1.13, 27.32 ± 5.56, 30.00 ± 7.48, and 37.63 ± 8.17 µg/mL, respectively. After exposure to HepG2 cells for twenty-four hours, the IC50 value for TL-MG was found to be 787.55 ± 12.78 µg/mL, showing that it is not harmful in any way.

### 2.13. Acute Toxicity Studies

Nine healthy albino rats weighing between 200 and 300 g were chosen and kept in a climate-controlled environment with access to food and water during the acute toxicity trial. Among these, three were kept in a group named the control, the other three were used for commercial products (Imunol), and the remaining three rats represented the third group named the TL-MG group. The quantity of TL-MG was equivalent to 10 mg/kg applied on the skin of (TL-MG group) rats. The control group was given water and food only. Biochemical findings on the fourteenth day of the study were noted (Table 3). For biochemical analysis, 1.0 mL of blood was drawn and sent to the laboratory for testing. The biochemical blood results are shown in Table 3. Neither any morbidity nor any mortality was noticed in any of the groups [42]. In addition, tests comparing the TL-MG group and the control group found no statistically significant differences in indices such as hemoglobin, red blood cells, white blood cells platelets, monocytes, and more. Minor changes in hematologic values were found between the groups, although all values were within normal limits.

### 2.14. Effect of TL-MG on IL-2 mRNA Expression Levels

**Figure 11:**
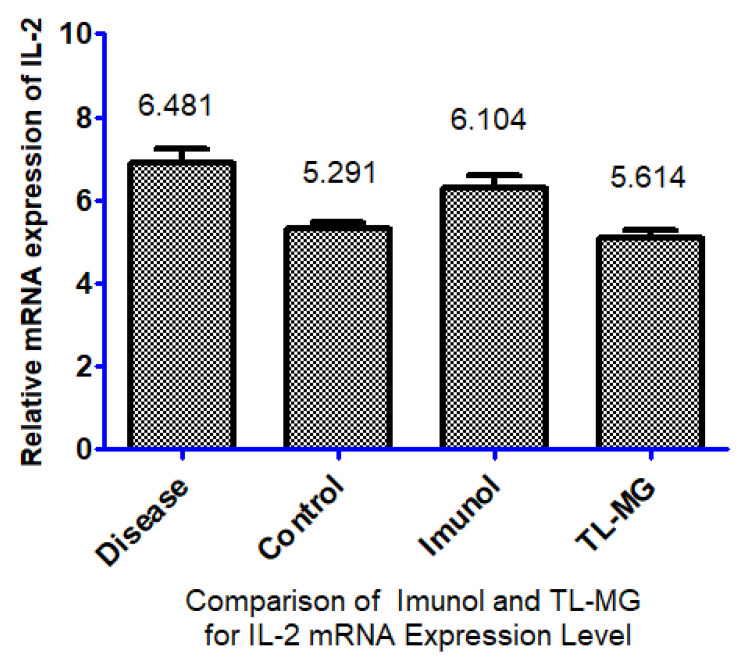
Effect of TL-MG on the IL-2 mRNA expression level.

According to the findings, the levels of IL-2 mRNA expression in the group that had the disease were significantly higher than those in the control group. When compared to the ill group, the levels of IL-2 mRNA expression were significantly lower in the group that received treatment with TL-MG and the Immunol commercial product. In this study, we used a total of four groups of animals. Every group, with the exception of the control group, was given an OVA solution through intravenous injection for 14 days. Fourteen days after being exposed to OVA, two groups started taking the medications Immunol and TL-MG, and one group continued with OVA solution for 28 days, which is known as the diseased group. According to the findings, the levels of IL-2 mRNA in both groups dropped significantly when we used Immunol and TL-MG. When compared to group I (control), the mRNA level of IL-2 in group II (disease) showed a considerable increase (6.4810.117 vs. 5.2910.131), as shown in Figure 11. The levels of IL-2 in group III treated with the commercial product Immunol did not demonstrate a significant decrease compared to the levels in diseased group II (6.1040.187 vs. 6.4810.117). Compared to diseased group II with IV, which had received TL-MG, there was a considerable decrease in the level of IL-2 (5.6140.113 vs. 6.4810.117).

## 3. Conclusions

It has been suggested that polymer-based methods for controlled drug delivery would predominate in the present and the future due to their many potential benefits for both scientific and commercial reasons. The idea behind the development of the polymeric microsponges delivery system was to distribute medications continuously for a long time to decrease the frequency of applications and hypersensitivity reactions and to increase bioavailability and safety compared to commercially available conventional formulations. TL-loaded microsphere gel presented an inimitable platform for topical delivery that is safe and simple to produce and designates the benefit of the desired release. The present study provided an innovative formulation of TL-MG for the treatment of severe atopic dermatitis caused by psoriasis. Microsponges that had been formed had a spherical shape, a high porosity, and good flow. The effect of different drug-polymer ratios on particle size, drug content, and encapsulation effectiveness was striking. The system was thermally stable and had a porous surface, which facilitated enhanced penetration of tacrolimus inside the system during drug loading. The system demonstrated no toxic effects and exhibited biological compatibility. In vitro studies on drug release and penetration exhibited a favorable profile and improved penetration compared to commercial products. TL-MG, initially synthesized for topical drug delivery, can now be utilized for controlled oral drug delivery as well, owing to the incorporation of bioerodible polymers in the synthesis of the delivery system. This synthesis is very simple and can be easily transferred for scale-up in pharmaceuticals. Very economical ingredients were used for its preparation in simple utensils.

## 4. Materials and Methods

### 4.1. Materials

Tacrolimus was a gift from Saffron Pharmaceuticals Pvt. Ltd., Pak, Faisalabad, Pakistan). Polyvinyl alcohol (Mw 89000) and Poloxamer 407 were purchased from Merck (Darmstadt, Germany). Tween 60 and ethanol were purchased from Daejung (Sihwa Industrial Complex 186 Seohaean-ro Sinan, Gyeonggi, 15087 Republic of Korea). Sodium hydroxide, Carbopol, and triethanolamine were purchased from Daejung (Sihwa Industrial Complex 186 Seohaean-ro Sinan, Gyeonggi, 15087 Republic Of Korea). Other chemicals used were also from Daejung, Seohaean ro, South Korea, and distilled water was taken from Saffron Pharmaceuticals Pvt limited. Millipore, located in Single Oak Drive Temecula California, United States, was the supplier of the Strat-MTM membranes. Strat-MTM has two layers of polyethersulfone and one layer of polyolefin, with the polyolefin layer sitting on top. Polymeric layers create a porous membrane with a gradient in pore size and diffusivity. This gradient is achieved through the layers. The synthetic membrane resembles skin due to its porous structure infused with a special blend of synthetic lipids. The membrane is 2.5 cm in diameter and 300 μm thick, while the filter has a 25 mm diameter.

### 4.2. Fabrication of Microsponges

The microsponges of TL were synthesized by a modified emulsion solvent evaporation technique [31]. In the organic phase, matrix-forming polymers such as poloxamer 407 and polyvinyl alcohol (Mw 89000) were dissolved in a 1:1 ratio (5.0 mL ethanol and 5.0 mL water were used as solvents) (polymeric phase). After this, we added 50 mg of TL into 5 mL of ethanol for solubilization and then added it to the internal polymeric phase. The ingredients were vortexed until a clear solution was achieved. Five milliliters of Tween 60 was added to 100 mL of distilled water with continuous stirring. Then, the internal polymeric phase was added to the Tween 60 solution using a high-speed homogenizer (500 rpm), and the temperature was kept at 45 °C. After 15 min, precipitation started, and ethanol was evaporated due to heat, which was confirmed by observing the total volume of solution. Porous microsponges were produced, which were filtered with a 0.45 micron filter (Whatman), dried at room temperature for 24 h, and named TL-M. This TL-M was added to an overnight-soaked (1 g-1% *w*/*v*) aqueous dispersion of Carbopol 940. For uniform mixing of the aqueous system, the mixture was magnetically agitated at 350 rpm for 24 h. After the mixture was mixed uniformly, 30 μL of 2% *v*/*v* triethanolamine was added to neutralize Carbopol 940, bringing the pH level closer to 7.0 ± 0.5, and the mixture was named TL-MG. In Table 4, the full formulation is mentioned.

### 4.3. Characterization of Microsponges

#### 4.3.1. Entrapment Efficiency and Yield

Isocratic high-performance liquid chromatography was carried out with a Shimadzu LC 2030C 3D Plus system equipped with a photodiode array (PDA) detector under ambient conditions. The column used for the analysis was a Shim-pack C18 column measuring 250 mm by 4.6 mm with a 5 μ pore size. The value of 220 nm was chosen for the wavelength [43]. To properly separate the analyte, the flow rate was maintained at 1 mL/min, and the total run time was set to 10 min. The mobile phase was made up of methanol and a sodium sulfate solution that had a ratio of 1:9 with sulfuric acid that had a concentration of 0.1. To conduct the analysis, 10 mg of microsponge dispersion was dissolved in 1 mL of dichloromethane (DCM) and then sonicated for a period of thirty minutes in an ultrasonic bath manufactured by Ultrawave in the United Kingdom. After thoroughly combining the solution with 1 mL of the mobile phase, it was centrifuged at a speed of 8000 revolutions per minute for fifteen minutes. The entrapment efficiency (in percent) was calculated with the help of an equation using the supernatant that was produced (1).

(1) $$Entrapment\;efficiency\left(\%\right)=\frac{Amount\;of\;drug\;present\;in\;particles}{Amount\;of\;drug\;used}\times 100$$

#### 4.3.2. Photon Correlation Spectroscopy (PCS)

Particle size and polydispersity index (PDI) were determined immediately after synthesis by following previously reported protocols [44]. For the purpose of determining the size, polydispersity index (PDI), and zeta potential of the microsphere dispersion, a Zetasizer from Malvern Instruments in the United Kingdom was employed. The data were recorded using a scattering angle of ninety degrees.

#### 4.3.3. FTIR Spectroscopy

An attenuated total reflection crystal cell was utilized in FTIR spectrometry, conducted using the Thermo Scientific Nicolet iN5 FTIR device (Waltham, MA, USA). A small amount of sample was spread over the crystal and pressed over the crystal using the metal tip. Spectra of TL, PVA, Tween 60, poloxamer, and TL-M were recorded in the range of 600–4000 cm^−1^ [26].

#### 4.3.4. Differential Scanning Calorimetry (DSC) Study

Pure TL, PVA, poloxamer, and TL-M were evaluated by differential scanning calorimetry. A DSC machine made by Perkin Elmer USA was used in this study. Standard empty aluminum crucibles with holes were utilized for scanning, and the temperature range was from 25 to 500 °C in a nitrogen atmosphere at a heating rate of 20 °C min^−1^ [45].

#### 4.3.5. Powder X-ray Diffraction (XRD)

X-ray powder diffraction analysis was performed on pure TL, PVA, poloxamer, and TL-M at the Bruker facility in Ettlingen and Leipzig, Germany. The subjected radiation was cobalt-filtered ferrous ions (Fe^2+^) with a wavelength of 1.7890 °A [46].

#### 4.3.6. Microscopic Study

A scanning electron microscope made by JEOL Ltd. in Tokyo, Japan, was used for the study. This was carried out so that the surface morphology of the synthesized TL-M could be examined. A two-sided adhesive tape was used to attach the TL-M formulation that had been created to metal stubs. It was gold-plated, dried in a vacuum, and then viewed at different magnifications with a scanning electron microscope [47]. Compound microscopy was used to determine TL-MG in gel form.

#### 4.3.7. Nitrogen Adsorption–Desorption Analysis

The surface area, pore volume, and size of standardized microsponges were calculated by nitrogen adsorption-desorption using a Gemini VII 2390 surface area analyzer (Micromeritics Instrument Corp., Norcross, GA, USA). The surface area was determined by nitrogen’s ability to bind to and release from the pores of the microsponges. Before beginning the analysis, the temperature was brought down to −196.15 °C, and the sample was degassed for twenty-four hours. Adsorption and desorption data were analyzed using both the Barret-Joyner-Halenda (BJH) and Brunauer–Emmett–Teller (BET) methodologies. This process was carried out to establish pore parameters [48].

#### 4.3.8. In Vitro Release Study

For the in vitro drug release investigation, a dialysis bag approach was chosen. The dried TL-M, which was equivalent to 10 mg of medication, was dispersed in 5 mL of dissolving media and then placed inside a dialysis bag that had an MWCO of 12–14,000 Daltons and was manufactured by by Medicell International Ltd. in the Liverpool, United Kingdom. Both ends of the bags were secured with PVC clips, and then they were individually suspended in 500 mL of acetate buffer with a pH of 4.5 and phosphate buffer with a pH of 5.5 [49]. Under constant stirring, the temperature was maintained at 37 °C ± 0.5. At intervals of time that had been previously set, samples of aliquots measuring 2 mL each were extracted and then replaced with an equal volume of new medium. The samples were centrifuged at a speed of 5000 revolutions per minute. After collecting and filtering the supernatant, we performed HPLC analysis [50]. The percentage release was calculated as a function of time [51]. The method was validated using a calibration curve (R^2^ = 0.998) for the quantification of the concentration of the drug in formulated samples.

#### 4.3.9. Kinetic Modeling

The pattern of drug release during the dissolution investigation from the TL-M formulation was estimated using the models that had previously been used to analyze the behavior of TL-M in vitro. This was performed so that the behavior of TL-M could be evaluated.

##### Zero Order

The zero-order approach elucidates the release of drugs from TL-M, which can be mathematically represented by Equation (2) [52]:

(2) $$Qt=Q_{0}+K_{0}t$$

where, in the context of therapeutic agent release, “*Qt*” represents the quantity of the therapeutic agent released within a given time “*t*”, while “*Q*_0_” denotes the initial quantity of the therapeutic agent present in TL-M. Additionally, “*K*_0_” refers to the rate constant.

##### First-Order

This model illustrates that the release of the active moiety from TL-M is dependent on the quantity of the drug present in the formulation. This relationship is represented by Equation (3) [53]:

(3) $$logC=logC_{0}-\frac{kt}{2.303}$$

Here, in the given context, “*C*_0_” represents the initial amount of the active moiety within TL-M, while “*k*” signifies the first-order rate constant. The slope of the equation is calculated as “−*k*/2.303.

##### Higuchi Model

This helps to explain why the release of the medicine coincides with the process of diffusion (Fick’s law) [51]. Equation (4) can be used to discuss the release from TL-M, which follows the Higuchi model’s release kinetics

(4) $$Ft=K^{2}\times t^{1/2}$$

where, in the context provided, “*Ft*” represents the quantity of the drug that remains undissolved, while “*K*^2^” refers to the Higuchi rate constant.

##### Hixson Crowell Model

The Hixson-Crowell model describes the releases from the systems where the change in surface area and changes in the diameter of the particles. The following Equation (5) will be used for calculation.

(5) $$Q_{0}^{1/3}\times Q_{t}^{1/3\;}=k\times t$$

where *Q*_0_ is the initial amount, *Q_t_* is the remaining amount at time *t*, and *K* is a constant.

##### Korsmeyer-Peppas Model

This kinetic tool is used to explain the release of the active moiety that has been entrapped in a polymeric system, and the explanation is stated by Equation (6) below [51].

(6) $$\frac{Mt}{M\infty }=Ktⁿ$$

Here, *Mt*/M∞ = *Mt* and M∞ are the cumulative amounts of drug released at time t and at infinite time. *K* = model release rate constant.

Fickian diffusion and non-Fickian diffusion will be pursued by the release of the therapeutic moiety depending on whether the diffusion exponent, n, is less than 0.45 or falls between 0.45 and 0.89. When n is greater than 0.89, the release process will follow case-2 transport, and when n is less than 0.89, it will follow super case-2 transport [54].

#### 4.3.10. Physical Appearance, Viscosity, Spreadability, and pH Determination of TL-MG

The microsponges that were integrated into the gel were analyzed for their physical characteristics. To determine the pH, a digital pH meter (Inolab WTW 730, New York, NY, USA) was utilized, and a 1% TL-MG dispersion that was created in a medium consisting of water was tested. The viscosities of the formulations were assessed at 0.5, 1, and 2 rpm using a Brookfield viscometer (Model DV-II) with spindle 21. Spreadability was also evaluated as a glass slide marked earlier for a 1.0 cm circle, and 0.5 g of TL-MG was placed in the center of that circle and pressed with a second glass slide of the same size (500 g weight) on top of the upper slide for approximately 5 min to spread the gel. The increase in the diameter was measured.

#### 4.3.11. In Vitro Diffusion Studies of TL-MG

The Franz diffusion cell was utilized during the course of this experiment. The permeation of TL-MG was evaluated using a membrane (Strat-M^TM^ membranes) fitted into a vertical Franz diffusion cell (SES Analytical-Systems-GmbH, Bechenheim, Germany). Parafilm was used to enclose the sample on the donor side. Buffers of varying pH levels (4.5 and 5.5) were pumped through a silica membrane into the receptor chamber. Using a water circulator, the heat was maintained at 37 °C ± 0.5. Aliquots of 0.5 mL were taken from the receptor side at regular intervals, and then they were analyzed by HPLC at a wavelength of 220 nm. After that, an equal volume of newly made buffer was added to the receptor compartment [55].

#### 4.3.12. In Vitro Cell Viability Studies of TL-MG

The human hepatocellular (HepG2) cell line was originally obtained from the University of Lahore and is presently maintained by the American Type Culture Collection (ATCC). The cell culture method was optimized using Dulbecco’s modified Eagle’s medium (DMEM). Cells were cultured in this medium supplemented with 10% *v*/*v* fetal bovine serum at 37 °C in a moistened environment containing 5% carbon dioxide [56]. The cells were seeded for the first time the day before they were scheduled to be cultured with TL-M. To measure the degree of succinate dehydrogenase activity in the mitochondria, an MTT (3-(4,5-dimethylthiazol-2-yl)-2,5-diphenyltetrazolium-bromide) assay was carried out. This was conducted as a measurement of both the cells’ ability to survive and their capacity to proliferate. After a period of 24 h, different concentrations of TL-M ranging from 50 to 400 μg/mL were added to the medium containing the cells. After that, a final concentration of 5 mg/mL MTT diluted with phosphate-buffered saline (PBS) was applied to each well using 15 μL of the MTT solution. After incubation for four hours at 37 °C, the medium in each well was withdrawn carefully, and then it was replaced with 100 μL of dimethyl sulfoxide (DMSO), which dissolved the formazan crystals that had developed in the well. This process was repeated until all of the crystals in the well had dissolved. Microplate reader absorbance readings were taken at 570 nm, and the proportion of viable cells in each well was calculated using Equation (7) [57].

(7) $$\%\;Cell\;Viability=\frac{Abs\;of\;treated\;cell-Abs\;of\;blank}{Abs\;of\;Control-Abs\;of\;Blank}\times 100$$

#### 4.3.13. Hemolytic Investigations of TL-MG

Following the collection of rat blood in an EDTA-containing tube, the sample was centrifuged at a speed of 1500 revolutions per minute for a duration of five minutes. The speed was kept constant during the entire process. Phosphate buffer saline (PBS) was used in a washing procedure that consisted of three stages after the supernatant had been removed from the precipitate. Washed blood sediment (200 μL) was added to phosphate buffer saline (3.8 mL), and the combination was vortexed for a few minutes. After that, samples of varying quantities were added, and the mixture was maintained at 37 °C for two hours before being centrifuged for five minutes at a speed of 1600 revolutions per minute. Note the absorbance of the supernatant at 541 nm. For the purpose of this investigation, Triton-X served as a positive control. Through the use of microscopy, abnormalities in the blood cells were noticed, and the percentage of hemolysis (Equation (8)) was calculated [58].

(8) $$Hemolysis\left(\%\right)=ABS-\frac{{ABS}_{0}}{ABS100}-{ABS}_{0}\;\times 100$$

#### 4.3.14. Acute Toxicity Studies of TL-MG

According to the guidelines set by the Organization for Economic Co-operation and Development (OECD), the toxicity of the TL-MG formulation was evaluated in an animal model. All experimental methods were authorized by the Ethical Review Board of the Rashid Latif College of Pharmacy (RLCP) and given the Identifier RLCP/EP/2022 by the Institutional Review Board. Furthermore, all of the experiments were performed in line with the Declaration of Helsinki, which was issued by the World Medical Association. In the animal house at Lahore University, for a period of two weeks, six albino rats weighing between 150 g and 170 g each were separated into two groups: the testing group and the control group. The test group received TL-M by topical application. On the fourteenth day, blood samples were drawn and analyzed with regard to several biochemical characteristics [59].

#### 4.3.15. Determination of Proinflammatory Cytokine IL-2 mRNA Expression

To measure how much of the proinflammatory cytokine IL-2 is expressed in skin tissues, we performed a reverse transcription polymerase chain reaction (RT–PCR). All experiments were conducted in compliance with the World Medical Association’s Declaration of Helsinki and were authorized by the Rashid Latif College of Pharmacy’s (RLCP) Ethical Review Board (IRB No. RLCP/EP/90/2022). Rats were used for this experiment and divided into four groups. All groups were exposed to ovalbumin. OVA (20 μg) was dissolved in phosphate buffer and administered intraperitoneally (I.P.) to all groups, with the exception of the control group, at days 0 and 14. After 14 days, one group was treated with Immunol, the second group was treated with TL-MG, the third group was not treated and continued to be injected with OVA solution, and the fourth group was controlled without exposure to O. The standard TRIzol method was used for the extraction of total RNA from blood. Blood samples (200 μL) were combined with 600 μL of TRIzol reagent, followed by the addition of chloroform for phase separation and isopropanol for RNA precipitation. The recovered RNA was washed with ethanol, allowed to air dry, and then stored (−80 °C). cDNA was synthesized by reverse transcription. After thoroughly combining the RNA template and the primer, nuclease-free water (q.s.) was then added. After completion of the chemical processes, the mixture was subjected to a second round of incubation at 40 °C for 60 min.

## Figures and Tables

**Table 1:** The fitted equations of TL-M.

Models	Fitted Equation *	R^2^
Zero order	Q = 4.2863t + 4.5843	0.9429
First order	Q = −0.1209t + 0.0813	0.8091
Higuchi	Q = 26.783t^1/2^ − 23.2163	0.8931
Korsmeyer-Peppas	Q = 7.3821t^0.85^ − 2.0263	0.9650
Hixson Crowell	(1 − Q)^1/3^ = 0.03819t + 0.9107	0.8732

* Q is the cumulative drug released.

**Table 2:** Tropical gel formulation parameters.

Formulation	Clarity	pH	Homogeneity	Viscosity (Cpi)
TL-MG	Clear	5.5	Good	8712
Commercial product	Clear	6.1	Good	5235

**Table 3:** Biochemical blood analysis.

Parameters	Group 1	Group 2	Group 3
	(TL-MG)	(Control)	(Imunol)
Hb (10–15) g/dL	11.8 ± 0.98	12.2 ± 0.78	12.3 ± 1.21
WBCs × 10^9^/L	6.2 ± 0.45	5.2 ± 0.98	6.5 ± 0.24
RBCs × 10^6^/mm^3^	5.42 ± 0.42	5.76 ± 0.78	5.99 ± 1.11
Platelets × 10^9^/L	248 ± 8.90	286 ± 8.90	272 ± 9.35
Monocytes (%)	03 ± 0.05	07 ± 0.78	09 ± 0.78
Neutrophils (%)	40 ± 2.98	40 ± 1.26	56 ± 1.26
Lymphocytes (%)	52 ± 7.39	50 ± 2.90	60 ± 2.78
Eosinophils	02 ± 0.021	03 ± 0.06	02 ± 0.04
Blood sugar random mg/dL	71 ± 0.85	52 ± 3.89	72 ± 2.89
Uric Acid (Serum)mg/dL	8.4 ± 0.34	5.96 ± 1.08	7.9 ± 1.21

**Table 4:** Composition of the microsponge formulation.

Ingredients	Quantities
TL (mg)	50
Poloxamer (mg)	100
PVA (mg)	100
Tween 60 (mL)	5
Triethanolamine (mL)	Q.S
Ethanol (mL)	10
Carbopol (g)	1
Distilled water (mL)	100

## Data Availability

Not applicable.
